# Genetic Regulation of Protein Biomarkers in Pediatric Acute Respiratory Distress Syndrome

**DOI:** 10.1097/CCE.0000000000001468

**Published:** 2026-08-17

**Authors:** Guy Helman, Rui Feng, Joshua E. Motelow, Nadir Yehya

**Affiliations:** 1 Department of Anesthesiology and Critical Care, Children’s Hospital of Philadelphia and University of Pennsylvania, Philadelphia, PA.; 2 Department of Biostatistics, Epidemiology, and Informatics, Center for Clinical Epidemiology and Biostatistics, University of Pennsylvania, Philadelphia, PA.; 3 Division of Critical Care and Hospital Medicine, Department of Pediatrics, Vagelos College of Physicians and Surgeons, Columbia University, New York, NY.; 4 Leonard Davis Institute of Health Economics, University of Pennsylvania, Philadelphia, PA.

**Keywords:** acute respiratory distress syndrome, biomarker, genotyping, pediatrics

## Abstract

**OBJECTIVES:**

Acute respiratory distress syndrome (ARDS) is a significant contributor to ICU admissions, morbidity, and mortality. Biomarkers have increasingly been used to dissect heterogeneity and mechanisms underlying pediatric ARDS. We sought to assess the genetic contribution to the levels of protein biomarkers currently used to subphenotype pediatric ARDS patients.

**DESIGN, SETTING, AND PARTICIPANTS:**

Retrospective, single-center cohort study at a tertiary care PICU of children (< 18 yr) with pediatric ARDS diagnosis.

**INTERVENTIONS:**

None.

**MEASUREMENTS AND MAIN RESULTS:**

Genome-wide protein quantitative trait loci (pQTL) analysis was performed on biomarkers measured on day 0 of ARDS, which were previously associated with outcomes from a single-center cohort using linear regression models. We then assessed the genetic association with longitudinal biomarker levels (on days 0, 3, and 7) and with 28-day survival using linear mixed-effects models and Cox proportional hazard models, respectively. From 333 children with ARDS, 267 underwent pQTL analysis against 6 preselected protein biomarkers. Among the patients, 49.4% had ARDS attributed to infectious pneumonia, and 21.7% had ARDS resulting from nonpulmonary sepsis. The hypoinflammatory phenotype was identified in 74.2% of patients, and overall mortality was 14.6%. One single nucleotide polymorphism (SNP) was identified in *SFTPD* with presumed cis-regulatory effect. Linear mixed-effect modeling confirmed the effect of the SNP on biomarker levels in heterozygous or homozygous carriers on day 0 of ARDS course. Survival analysis demonstrated no significant impact on mortality risk.

**CONCLUSIONS:**

We identified a gene-associated SNP in *SFTPD* that impacts relevant biomarker levels in pediatric ARDS. Further characterization is needed to better understand subphenotypes in pediatric ARDS, including genetic determinants of the biomarker levels used to define them.

**KEY POINTS**

**Question**: Biomarker profiling acute respiratory distress syndrome (ARDS) has permitted stratification of patients into hypo- and hyperinflammatory subtypes with prognostic and predictive relevance. We sought to assess the genetic contribution to protein biomarkers currently used to subphenotype pediatric ARDS patients.

**Findings**: We identified a single nucleotide polymorphism in *SFTPD* that was associated with reduced surfactant protein D levels in pediatric ARDS.

**Meaning**: This study serves as a proof of concept for the potential utility of protein quantitative trait loci analysis to dissect the influence of genetic variation in a complex pediatric critical illness syndrome.

Acute respiratory distress syndrome (ARDS) is a heterogeneous condition of inflammatory lung injury and impaired oxygenation, contributing to ICU morbidity and mortality. Biomarker profiling in adult (1–3) and pediatric ARDS (pARDS) (4, 5) has permitted stratification of patients into hypo- and hyperinflammatory subtypes with prognostic and predictive relevance.

ARDS is typically caused by diverse triggers (e.g., pneumonia, sepsis), and most subjects exposed to these triggers do not develop ARDS. Thus, interindividual susceptibility raises the question of what factors predispose patients to ARDS. Genome-wide association studies (GWASs) have been used to examine the role of common genetic variants in susceptibility and severity of adult ARDS, partly via their association with circulating proteins (6–8). Challenges to GWAS in ARDS include heterogeneity of inclusion criteria based on definition, precipitating factors, and appropriate control definition.

An alternative strategy for genetic investigation in pediatric critical illness is the study of genetically regulated intermediate phenotypes, such as protein biomarkers. Protein quantitative trait locus (pQTL) analyses focus on identifying genetic variants that influence protein levels, thereby offering increased statistical power, improved biological interpretability, and clearer mechanistic insight compared with disease-level association testing. This approach is particularly relevant in ARDS, where plasma biomarkers are widely used to define subphenotypes and reflect key inflammatory and tissue injury pathways (4, 5). We performed a genome-wide pQTL analysis of plasma protein biomarkers in pARDS patients to identify genetic variants associated with biomarker variability and explore associations with clinical outcomes.

## METHODS

### Cohort Description

**Figure 1.**
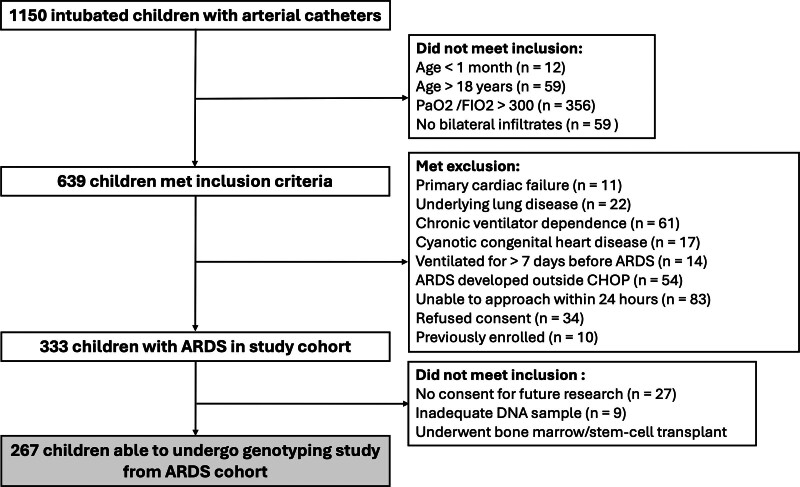
Pediatric acute respiratory distress syndrome (ARDS) cohort determination. Progression of ARDS cohort determination from initial screening through sample collection. Modified from Yehya et al (5) and Yehya et al (10). CHOP = Children’s Hospital of Philadelphia.

This was a planned secondary analysis of prospectively collected data. Patient recruitment and enrollment was performed under an Institutional Review Board (IRB) approved study at the Children’s Hospital of Philadelphia (CHOP IRB 13-010578; Biomarkers of Pediatric ARDS; approved July 2, 2014), conducted in accordance with the Helsinki Declaration of 1975. Informed consent was obtained prior to study procedures. As previously reported, intubated children with Berlin-defined ARDS (9) from the PICU were enrolled between 2014 and 2019 (**Fig. 1**) (5, 10). Samples from subjects consented for future research were used for this pQTL study. Individuals who underwent stem-cell transplant were excluded as their circulating leukocyte DNA would reflect donor cells. Inclusion criteria and full methodology are included in the **Supplemental Text** (https://links.lww.com/CCX/B674).

### Biomarker Measurements

The aim of the initial cohort was to determine the association of biomarkers with outcomes in pARDS. Biomarkers were selected based on previous studies and included angiopoietin 2, the soluble receptor for advanced glycation end-products, surfactant protein D (SPD), interleukin (IL)-8, IL-6, and soluble tumor necrosis factor receptor 1. Biomarkers were measured using a combination of single and multiplex enzyme-linked immunosorbent assays as previously described (11).

### DNA Extraction and pQTL Analysis

After DNA extraction from stored blood pellets, samples underwent genomic DNA cleanup, single nucleotide polymorphism (SNP) genotyping was performed using the Omni 2.5 platform (Illumina, San Diego, CA). We removed samples with greater than 5% missing genotypes, a minor allele frequency of less than 5%, and Hardy-Weinberg *p* values of less than 0.001. Principal component (PC) analysis was performed to examine outliers and capture genetic variation related to ancestry. Biomarker values were log-transformed for normality.

### Analysis

We performed pQTL analysis using linear regression for each biomarker after log-transforming day 0 biomarker values, assuming an additive genetic model implemented in PLINK v1.9.0 beta 7 (Harvard University, Cambridge, MA) (12). Models were adjusted for sex, age at ARDS, and the top three PCs to account for population stratification and admixture based on sensitivity analysis (Supplemental Text, https://links.lww.com/CCX/B674). Significance was determined using the Benjamini-Hochberg method to control the false discovery rate at a threshold of 5%. The number of PCs was determined by optimizing genomic inflation factors (λ; **Supplemental Figs. 1** and **2**, https://links.lww.com/CCX/B674). Because this study was designed to identify genetically regulated variation in disease-relevant proteins rather than ARDS susceptibility, we prioritized SNPs with *p* < 1 × 10^–7^ for downstream analyses. We then adjusted for Pediatric Risk of Mortality (PRISM) and ARDS subphenotype (5).

We tested the association of selected SNPs with log-transformed longitudinal biomarker levels over time using linear mixed-effects models (13), adjusted for sex, age, and the three top PCs as fixed effects, and a subject-specific random effect to account for within-subject correlation. Nonproportional hazards were assessed using Schoenfeld residuals. A time-varying Cox model was fit, allowing the effect of carrier status to vary with log(time).

## RESULTS

Two hundred sixty-seven individuals with ARDS underwent genotyping and were included in our pQTL analysis (Fig. 1). Among the patients, 49.4% had ARDS attributed to infectious pneumonia (**Supplemental Table 1**, https://links.lww.com/CCX/B674). Additionally, 12.4% of patients were immunosuppressed. Of this cohort, 74.2% were classified in the hypoinflammatory subphenotype (5). Overall mortality was 14.6%. The cohort was racially and ethnically diverse, with 44.9% identifying as White, 29.2% as Black, and 11.6% as Hispanic.

pQTL analysis was performed on six biomarkers using day 0 log-transformed values adjusted for age, sex, and three PCs, as well as clinical severity using the PRISM score and assigned subphenotype. The association between rs2146192 and plasma SPD remained essentially unchanged after adjustment for age, sex, ancestry, illness severity, and inflammatory subphenotype (**Supplementary Table 2**, https://links.lww.com/CCX/B674). We assessed available population allele frequency date for discrepancies (**Supplemental Table 3**, https://links.lww.com/CCX/B674), prioritizing one SNP for further analysis. rs2146192 was identified in *SFTPD* and the C allele was associated with decreased levels of SPD (**Supplemental Tables** 3 and **4**, https://links.lww.com/CCX/B674). *SFTPD* encodes SPD suggesting a cis-regulatory effect. Of 71 individuals with at least one copy of the C allele, 25% were classified as the hyperinflammatory subphenotype, and there was 11% mortality (Supplemental Table 4, https://links.lww.com/CCX/B674).

**Figure 2.**
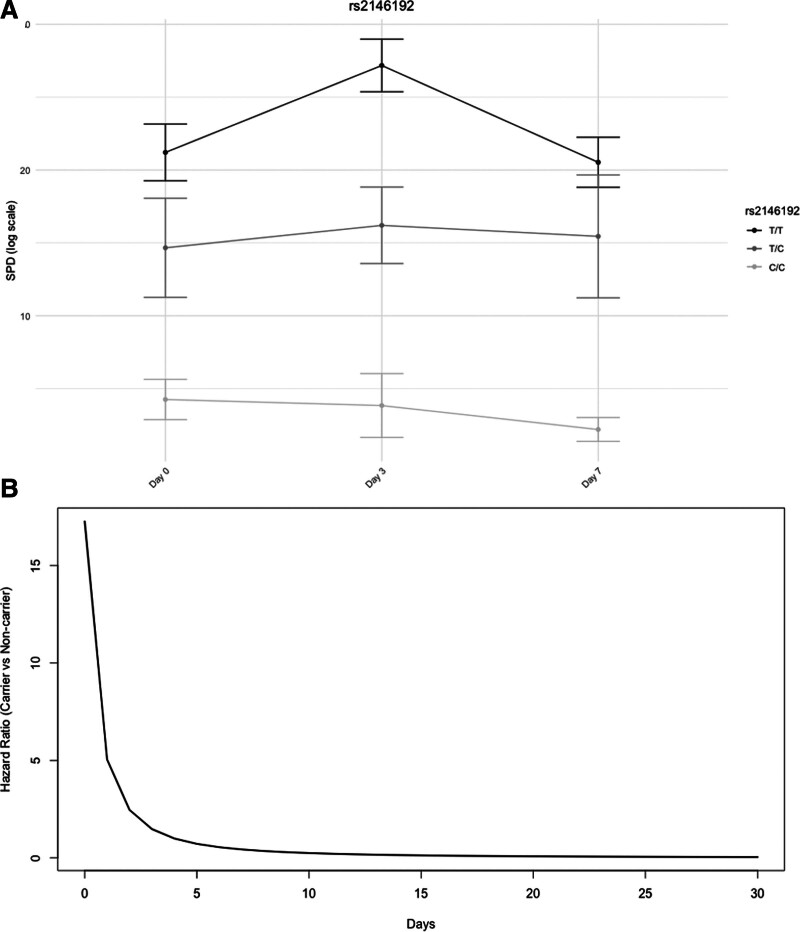
Longitudinal variation in biomarker levels by genotype and time-dependent association of mortality with rs2146192 C allele. *Plots* were made using nontransformed data. **A**, Individuals with one and two copies of rs2146192 C allele showed lower baseline surfactant protein D (SPD) levels (estimate = –0.56, *t* = –3.82; estimate = –1.45, *t* = –4.35, respectively). **B**, In time-varying Cox models allowing the genotype effect to vary with time, rs2146192 demonstrated a significant time-dependent association with mortality, characterized by increased early hazard with attenuation and reversal over time (time-interaction *p* = 0.036).

Linear mixed-effect modeling was performed using rs2146192 C allele and longitudinal SPD data points to understand the contribution of genotype to biomarker levels over time (**Supplemental Table 5**, https://links.lww.com/CCX/B674). rs2146192 C allele appeared to play a role in SPD levels, but not trajectory (**Fig. 2*A***). Individuals with one and two copies of rs2146192 showed lower baseline SURPFD levels (estimate = –0.56, *t* = –3.82 for T/C; estimate = –1.45, *t* = –4.35 for C/C). However, the interaction terms between genotype and time (days 3 and 7) were not statistically significant, suggesting the rs2146192 C allele primarily influences baseline biomarker expression rather than longitudinal time-dependent SPD levels.

In Cox models, rs2146192 C allele carrier status was not independently associated with mortality (hazard ratio [HR], 0.61; 95% CI, 0.27–1.40; *p* = 0.27). However, assessment of proportional hazards assumptions demonstrated significant nonproportionality for carrier status, indicating that the HRs may vary over time. We used a time-varying Cox model, allowing the genotype effect to vary with time, where the rs2146192 C allele demonstrated a time-dependent association with mortality, characterized by increased early hazard with attenuation and reversal over time (time-interaction *p* = 0.036; **Fig. 2*B***).

## DISCUSSION

We identified the rs2146192 C allele in *SFTPD* as a cis-pQTL associated with lower circulating SPD in pARDS. While these results warrant replication in additional cohorts, this initial pQTL study may shed light on the genetic risk factors associated with development of ARDS, biological pathways implicated in ARDS, and potential predictors of clinical outcomes after ARDS.

The C allele at rs2146192 is an intronic SNP previously identified in a GWAS cohort of individuals with chronic obstructive pulmonary disease, where individuals with this SNP had depressed levels of SPD (14) and as a cis-pQTL in a proteogenomic study in European individuals (15). Previous studies have shown that SPD elevation is associated with severe pARDS and poor outcomes, including prolonged need for mechanical ventilation, PICU length of stay, and mortality (16), and that, in nonsurvivors, increases over the initial 7 days of the ARDS course (11). However, SPD has not been shown to reliably distinguish biomarker-based hypo- and hyperinflammatory subphenotypes in adult ARDS or pARDS (4, 5).

In our study, rs2146192 C allele was a cis-pQTL associated with lower circulating SPD levels. While SPD exhibited significant temporal variation following ARDS onset, there was no evidence that genotype modified the longitudinal trajectory, suggesting that rs2146192 C allele primarily influenced baseline SPD expression rather than time-dependent regulation. In prior studies (16), higher plasma SPD was associated with increased mortality and greater disease severity. However, rs2146192 carrier status was not independently associated with mortality, and there was no evidence that plasma SPD mediated a genetic effect on survival. Time-varying Cox models suggested a transient early association with mortality, likely reflecting nonproportional hazards rather than causal genetic effects. Taken together, these data suggest a model where rs2146192 C allele regulates baseline SPD levels but does not directly drive disease progression or pARDS mortality risk.

While a strength of our study is the sample size relative to other pediatrics ARDS studies with DNA collection, the sample size is small and likely underpowered for full genotypic analysis. GWAS studies in adult ARDS were larger and better powered for genotypic association. The intrinsic heterogeneity of ARDS further limits clean genotype-driven associations with biomarkers and with outcomes, as does population admixture and having diverse cohorts in our pediatric setting. Confounder adjustment only partly mitigates this given contributing genetic, environmental, and disease factors. Preclinical models will help further understand the functional consequences of the observed SNP. However, our results implicate potential pathways for pARDS via a biologically plausible cis-pQTL and provide evidence for genetic lineage as a source of heterogeneity. While there has been limited use of GWAS methods in adult ARDS (6–8), there was no direct replication of findings from adult ARDS studies in our pediatric cohort.

Given the limited feasibility of using large-scale GWAS in a small pediatric critical illness cohort, we used a pQTL-based approach to focus on genetically regulated variation in disease-relevant proteins. Our identified SNP genetically regulates SPD levels, thereby implicating genetic control of a biologically plausible driver of pathology in pARDS. Given that no independent validation cohort is currently available, we recognize that these findings are exploratory. Further characterization is needed to better understand genotypic association with biomarkers and subphenotypes in pARDS and their potential contribution to improved biological understanding and future therapeutic development in this condition.

## ACKNOWLEDGMENTS

We thank the patients and their families.

## Supplementary Material


