# Supplementary material for: Genetic Regulation of Protein Biomarkers in Pediatric Acute Respiratory Distress Syndrome
Source: Crit Care Explor. 2026 Aug 17;8(8):e1468. doi: 10.1097/CCE.0000000000001468 (PMC13485844; doi:10.1097/CCE.0000000000001468)
Supplement: Supplementary file 1 [file cc9-8-e1468-s001.pdf]

Supplemental Text: Genetic regulation of protein biomarkers in pediatric acute respiratory distress syndrome

Guy Helman MD, PhD[1], Rui Feng PhD[2], Joshua E Motelow MD, PhD[3], Nadir Yehya MD, MSCE[4,5]

Affiliations:
1. Department of Pediatrics, Children's Hospital of Philadelphia, Philadelphia, PA
2. Department of Biostatistics, Epidemiology, and Informatics, Center for Clinical Epidemiology and Biostatistics, University of Pennsylvania, Philadelphia, PA
3. Division of Critical Care and Hospital Medicine, Department of Pediatrics, Vagelos College of Physicians & Surgeons, Columbia University, New York, NY
4. Department of Anesthesiology and Critical Care, Children's Hospital of Philadelphia and University of Pennsylvania, Philadelphia, PA
5. Leonard Davis Institute of Health Economics, University of Pennsylvania, Philadelphia, PA

Corresponding Author: Nadir Yehya
E-mail Address: YEHYAN@chop.edu

<u>Supplemental Methods:</u>
Methods

<u>Cohort Description</u>

This was a planned secondary analysis of prospectively-collected data. Patient recruitment and enrollment was performed under an Institutional Review Board (IRB) approved study at the Children's Hospital of Philadelphia (CHOP IRB 13-010578; Biomarkers of Pediatric ARDS; approved July 2, 2014), conducted in accordance with the ethical standards set forth by the CHOP IRB consistent with the Helsinki Declaration of 1975. Informed consent was obtained prior to study procedures. As previously reported, intubated children with Berlin-defined ARDS (13) from the PICU were enrolled between 2014 and 2019 (Figure 1 (9, 14)). Inclusion criteria were 1) acute respiratory failure requiring invasive ventilation, 2) arterial access, 3) age > 1 month and < 18 years, 4) two consecutive $PaO_2/FIO_2 \leq 300 \geq 1$ hour apart on positive end-expiratory pressure (PEEP) ≥ 5 cmH_2O, and 5) new bilateral parenchymal infiltrates. Exclusion criteria were 1) respiratory failure primarily from cardiac failure, 2) underlying chronic respiratory disease, 3) ventilator dependence, 4) mixing cyanotic heart disease, 5) ventilation for > 7 days before $PaO_2/FIO_2 \leq 300$, and 6) ARDS established outside of the CHOP PICU. For this pQTL study, we only used samples from subjects that consented for future research. Individuals who underwent stem-cell transplant were excluded from further analysis as their circulating leukocyte DNA would reflect donor cells.

<u>Biomarker Measurements</u>

The aim of the initial cohort was to determine the association of biomarkers with outcomes in pediatric ARDS. After the first 54 subjects in whom plasma was collected within 24 hours of ARDS diagnosis, we expanded to additional plasma collection on day 3 and 7 of after ARDS onset (9, 15, 16) due to a change in study protocol. Biomarkers were measured using a combination of single and multiplex enzyme-linked immunosorbent assays (ELISAs). Angiopoietin 2 (ANG2), the soluble receptor for advanced glycation end-products (sRAGE), and surfactant protein D (SPD) were measured using singleplex ELISAs (Abcam, R&D Systems, and Abbexa). Interleukin-8 (IL-8) was measured on a Luminex platform (17). IL-6 and soluble tumor necrosis factor receptor 1 (sTNFR1) were measured using a custom Ella (Biotechne) multiplex assay.

<u>DNA Extraction and pQTL analysis</u>

After DNA extraction from stored blood pellets, samples underwent genomic DNA cleanup (Takara), quality control (Nano QC, Qubit QC, and Gel QC) and genotyping of 327,348 single nucleotide polymorphisms (SNPs) using the Omni 2.5 platform (Illumina). Quality control metrics to remove samples with >5% missing genotypes, minor allele frequency less than 5% and Hardy-Weinberg *P* values less than 0.001. Principal component (PC) analysis was performed to examine outliers and capture genetic variation related to ancestry. Biomarker values were log-transformed for normality.

Analysis

We performed pQTL analysis of each SNP with log-transformed biomarker values on day 0 using linear regression, assuming an additive genetic model implemented in PLINK v1.9.0 beta 7 (18). Models were adjusted for sex, age at ARDS, and the top three PCs to account for population stratification. Statistical significance was determined using the Benjamini-Hochberg method to control the false discovery rate (FDR) at a threshold of 5%. The number of PCs was determined by optimizing genomic inflation factors (λ) (Supplemental Figure 1-2). Because this study was designed to identify genetically regulated variation in disease-relevant proteins rather than ARDS susceptibility, we prioritized SNPS with strong biomarker associations for downstream analyses. We adjusted for PRISM and ARDS subphenotype (9). SNPs with $p < 1x10^{-7}$ were further evaluated.

We then tested the association of selected SNPs with log-transformed longitudinal biomarker levels over time using linear mixed-effects models (19), adjusted for sex, age, and the three top PCs as fixed effects, and a subject-specific random effect to account for within-subject correlation. Non-proportional hazards were assessed using Schoenfeld residuals. A time-varying Cox model was fit, allowing the effect of carrier status to vary with log(time).

Supplemental Tables and Figures:

Supplemental Table 1: Clinical characteristics stratified by participant survivorship

	Survival (N=228)	Death (N=39)	Overall (N=267)
**Age**	5.1 [1.7, 13.0]	6.8 [1.4, 12.4]	5.4 [1.6, 13.0]
**Sex**			
F	100 (43.9%)	18 (46.2%)	118 (44.2%)
M	128 (56.1%)	21 (53.8%)	149 (55.8%)
**Diagnosis**			
Infectious Pneumonias	127 (55.7%)	5 (12.8%)	132 (49.4%)
Sepsis	41 (18.0%)	17 (43.6%)	58 (21.7%)
Aspiration Pneumonia	35 (15.4%)	7 (17.9%)	42 (15.7%)
Other	25 (11.0%)	10 (25.6%)	35 (13.1%)
**Immunosuppressed**	22 (9.6%)	11 (28.2%)	33 (12.4%)
**Prism III at 12 hours**	9.0 [5.0, 14.2]	18.0 [14.5, 29.0]	11.0 [6.0, 17.0]
**Neurologic Failure**	32 (14.0%)	22 (56.4%)	54 (20.2%)
**Cardiovascular Failure**	131 (57.5%)	34 (87.2%)	165 (61.8%)
**Kidney Failure**	47 (20.6%)	24 (61.5%)	71 (26.6%)
**Liver Failure**	39 (17.1%)	27 (69.2%)	66 (24.7%)
**Hematologic Failure**	50 (21.9%)	23 (59.0%)	73 (27.3%)
**ARDS Subhenotype**			
Hypoinflammatory	182 (79.8%)	16 (41.0%)	198 (74.2%)
Hyperinflammatory	46 (20.2%)	23 (59.0%)	69 (25.8%)
**Ethnicity**			
Asian/Pacific Islander	9 (3.9%)	3 (7.7%)	12 (4.5%)
Black	70 (30.7%)	8 (20.5%)	78 (29.2%)
Hispanic	27 (11.8%)	4 (10.3%)	31 (11.6%)
Other	20 (8.8%)	6 (15.4%)	26 (9.7%)
White	102 (44.7%)	18 (46.2%)	120 (44.9%)

Supplemental Table 2: Association of rs2146192 in *SFTPD* with plasma SPD levels across alternative covariate specifications

Model	Covariates	A1/A2	Num	EAF	BETA	SE	P value
Base	Age, sex, PCs	C/T	267	0.1517	-0.7830	0.1332	1.28e-08
+PRISM3	Base + PRISM	C/T	267	0.1517	-0.7827	0.1335	1.38e-08
+Subphenotype*	Base + subphenotype	C/T	267	0.1517	-0.7869	0.1327	9.63×10^{-9}

A1: Effect allele; A2: Alternate allele; Num – Number of individuals testing; EAF: Effect allele frequency; SE: Standard error; *Subphenotype indicating hyperinflammatory or hypoinflammatory

Supplemental Table 3: Population allele frequencies of GWAS identified SNPs

rsID	Variant^	Genome AF	Homozygotes	European Finnish AF	South Asian AF	Admixed American AF	East Asian AF	European non-Finnish AF	Ashkenazi Jewish AF	Remaining AF	Middle Eastern AF	African/African American AF	Amish AF
rs2146192	10-79955982-T-C	0.1466	2068	0.06166	0.3052	0.2195	0.4105	0.1009	0.1504	0.1581	0.1667	0.1658	0.05482

AF – Allele frequency; ^ denotes variant using coordinates in GRCh38

Supplemental Table 4: Clinical characteristics stratified by rs2146192

	T/T (N=196)	T/C (N=61)	C/C (N=10)
**Age**	5.9 [1.8, 13.0]	4.4 [1.3, 11.8]	2.8 [1.3, 4.5]
**Sex**			
F	84 (42.9%)	30 (49.2%)	4 (40.0%)
M	112 (57.1%)	31 (50.8%)	6 (60.0%)
**Diagnosis**			
Infectious Pneumonias	94 (48.0%)	32 (52.5%)	6 (60.0%)
Sepsis	46 (23.5%)	10 (16.4%)	2 (20.0%)
Aspiration Pneumonia	32 (16.3%)	10 (16.4%)	0 (0%)
Other	24 (12.2%)	9 (14.8%)	2 (20.0%)
**Immunosuppressed**	23 (11.7%)	8 (13.1%)	2 (20.0%)
**Prism III at 12 hours**	11.0 [6.0, 17.0]	9.0 [4.0, 16.0]	12.0 [11.0, 16.2]
**Neurologic Failure**	39 (19.9%)	15 (24.6%)	0 (0%)
**Cardiovascular Failure**	122 (62.2%)	37 (60.7%)	6 (60.0%)
**Kidney Failure**	51 (26.0%)	16 (26.2%)	4 (40.0%)
**Liver Failure**	47 (24.0%)	15 (24.6%)	4 (40.0%)
**Hematologic Failure**	55 (28.1%)	15 (24.6%)	3 (30.0%)
**ARDS Subphenotype**			
Hypoinflammatory	145 (74.0%)	47 (77.0%)	6 (60.0%)
Hyperinflammatory	51 (26.0%)	14 (23.0%)	4 (40.0%)
**PICU Mortality**			
Survival	165 (84.2%)	54 (88.5%)	9 (90.0%)
Death	31 (15.8%)	7 (11.5%)	1 (10.0%)
**Ethnicity**			
Asian/Pacific Islander	5 (2.6%)	5 (8.2%)	2 (20.0%)
Black	60 (30.6%)	13 (21.3%)	5 (50.0%)
Hispanic	19 (9.7%)	11 (18.0%)	1 (10.0%)
Other	20 (10.2%)	6 (9.8%)	0 (0%)
White	92 (46.9%)	26 (42.6%)	2 (20.0%)

Supplemental Table 5: Associations Between SNP Genotype and Longitudinal Biomarker Levels

Biomarker	SNP	Genotype	Estimate (Day 0)	t-value	Significant Time Interaction on Day 3 or Day 7
SPD	rs2146192	T/C	–0.564	–3.82	None determined
		C/C	–1.448	–4.35	None determined

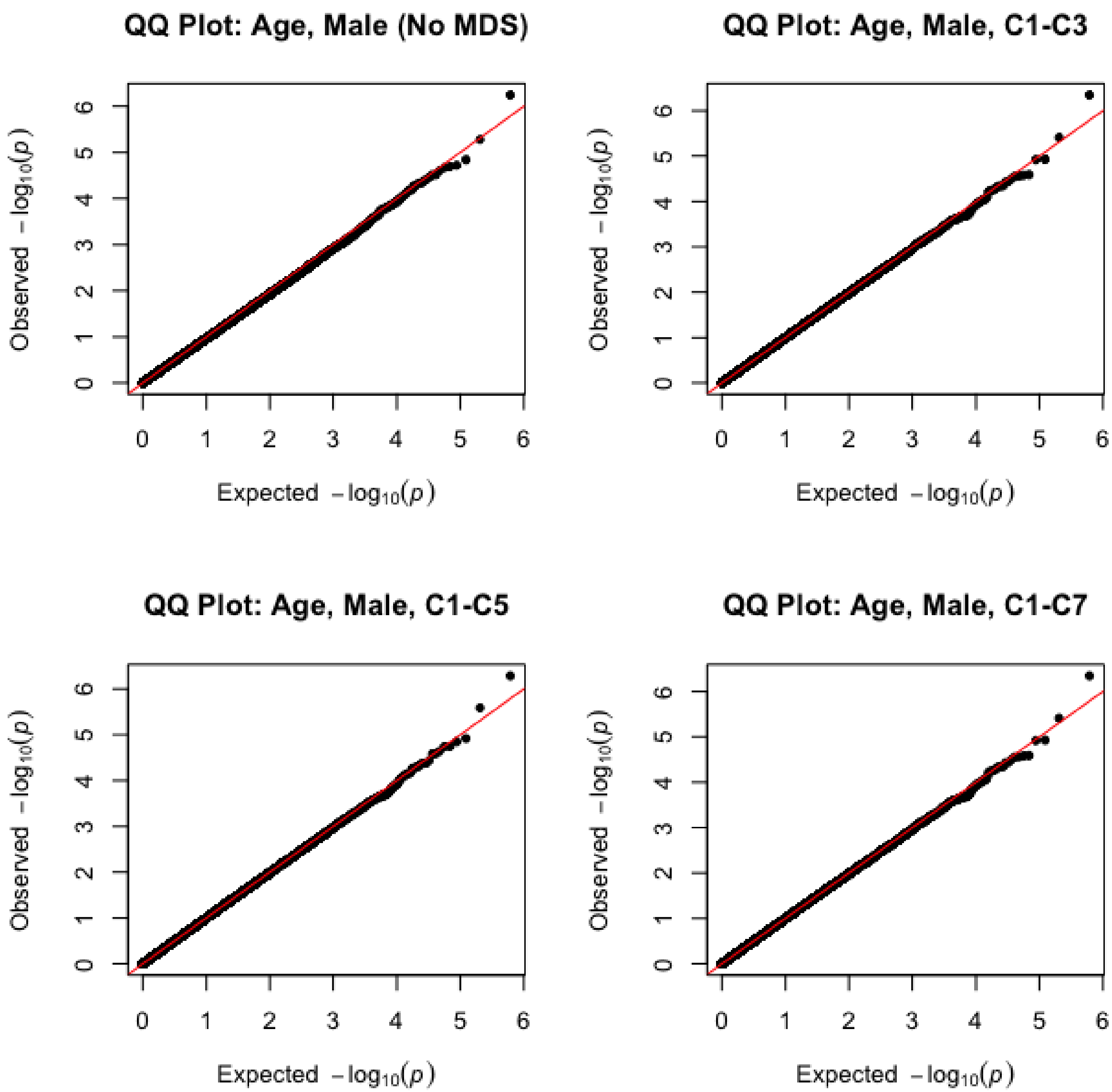

Supplemental Figure 1: Assessment of genomic inflation by number of covariates. Sensitivity analysis of genomic inflation based on the number of covariates demonstrates limited variability in correction based on the use of additional covariates.

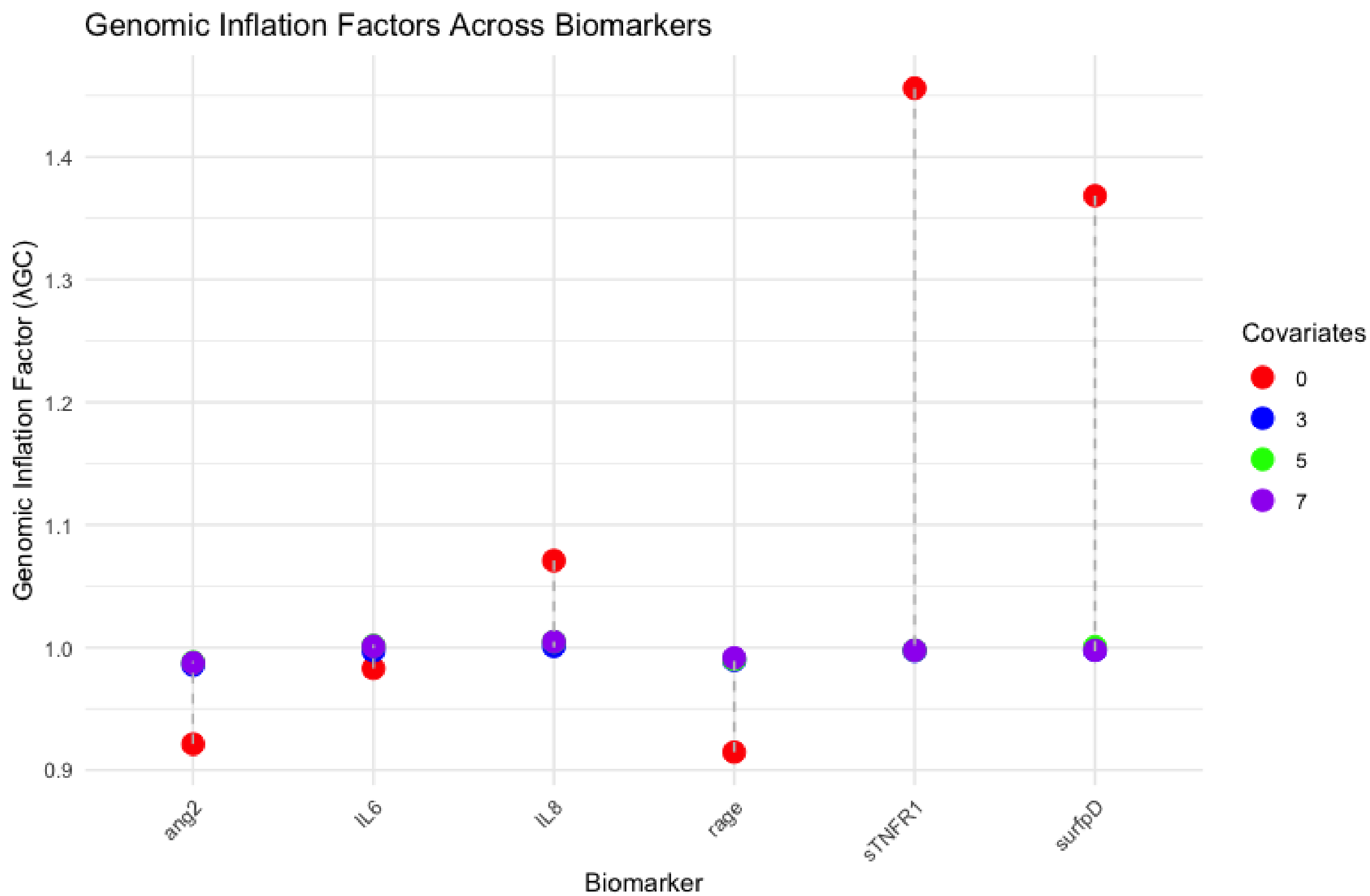

Supplemental Figure 2: Genomic inflation markers broken down by biomarker.
